# Preferences for Peer Support Amongst Families Engaged in Paediatric Screening Programmes: The Perspectives of Parents Involved in Screening for Type 1 Diabetes in Children Aged 3–13

**DOI:** 10.1111/hex.70007

**Published:** 2024-08-27

**Authors:** Ian Litchfield, Lauren M. Quinn, Felicity Boardman, Olga Boiko, Parth Narendran, Shivam Choundhary, Naga Setti, Veer Sheth, Sheila M. Greenfield

**Affiliations:** ^1^ Institute of Applied Health Research, College of Medical and Dental Sciences University of Birmingham Birmingham UK; ^2^ Institute of Immunology and Immunotherapy, College of Medical and Dental Sciences University of Birmingham Birmingham UK; ^3^ Division of Health Sciences University of Warwick Warwick UK; ^4^ Department of Diabetes The Queen Elizabeth Hospital Birmingham UK

**Keywords:** children and young people, long‐term conditions, peer support, population screening, Type 1 diabetes

## Abstract

**Introduction:**

This work describes a secondary analysis of a qualitative data set originally used to understand parent participants' preferences for the design and implementation of a screening programme for paediatric Type 1 diabetes (T1D). From this, their spontaneous preferences for peer support emerged, described here in the context of existing peer support programmes for the newly diagnosed alongside suggestions for their incorporation into screening programmes for T1D and a range of other conditions.

**Methods:**

Data were collected from semi‐structured interviews conducted with parents of children aged 3–13 years to explore their expectations, perceptions and preferences of a T1D paediatric screening programme. A secondary analysis of interviews from participants who spontaneously raised preferences for peer support was used to populate a novel framework informed by NHS England's key principles for the same, namely, *Shared experiences and reciprocated support*, Accessibility and inclusivity and *Person‐centred and integrated peer support*.

**Results:**

Parents in 29 of 33 interviews spontaneously described the potential value of peer support if receiving a result indicating a positive (presymptomatic T1D result) from a screening programme. Specifically, the value of ‘Shared experiences and reciprocated support’ in terms of emotional support and reassurance, and access to more directly interpretable and relevant information related to the condition; ‘Accessibility and inclusivity’ relating to access to a community of similar individuals, whether in person or online; ‘Person‐centred and integrated peer‐support’ and the need for support reflecting the changing need of the child and the integration of peer support with clinical care.

**Conclusions:**

The needs of peer support described by parents involved in T1D paediatric screening appear to be shared with those of families with children diagnosed with a range of life‐altering conditions. Although the needs of peer support for paediatric screening may differ across conditions, our findings are a valuable starting point for its design both in T1D and other examples of similar population screening programmes.

**Patient or Public Contribution:**

Patients and the public have been involved throughout the design of the ELSA study and have worked with us to inform the study process. They contributed to the design and content of patient‐facing materials, the content of our topic guides and the analysis and interpretation of our findings.

## 1 Introduction

Screening for long‐term and life‐altering conditions began at the turn of the twentieth century, though it took several decades before the availability of simple tests and effective clinical treatment meant it became more widespread [1, 2, 3]. Screening programmes have now become an established element of modern healthcare and are used for a range of common long‐term conditions, including cancer [4, 5, 6], heart disease [7] and a number of genetic conditions [8]. The benefits of understanding and preparing for the onset of illness or disease may be apparent but there are downsides; such programmes do not provide diagnoses with further testing often required [9]; they also induce anxiety in participants whether from the process of participation, the notification of positive results or the subsequent living with long‐term risk [10, 11]. The latter can be more pronounced where it is unknown when symptomatic onset is expected to occur or the precise consequences for the individual's life course [11, 12, 13, 14]. There are particular sensitivities in screening for children and young people (CYP), where not only should the needs of the participant be considered but also those of their family and carers who typically make the decision to participate [15, 16, 17]. As a result of the multiple and considerable impacts of screening, the UK's National Screening Committee produced a set of criteria to determine whether a screening programme should proceed based on the overall premise that it has the potential to achieve ‘more good than harm’ [18].

One independent source of assistance and understanding routinely sought in other areas of health care both for the newly diagnosed, or those otherwise assimilating life‐altering health conditions, is ‘peer support’ [19]. It is defined here as consisting of structured emotional support and the sharing of experience and practical learning from others living with a similar condition, facilitated by the National Health Service (NHS) [20]. Peers providing the support can be drawn from the same sociocultural background, those with the same condition or at its most effective a combination of both [19]. There is evidence it can improve health outcomes, enhance quality of life and alleviate psychological harm associated with the day‐to‐day challenges of managing long‐term conditions [20, 21]. Despite its potential in the context of UK screening, at the time of writing, there are currently no formal peer support interventions routinely offered to individuals identified as ‘at‐risk’” through population screening programmes in the United Kingdom [22].

If the benefits of peer support for those identified by screening as being ‘at risk’ are to be realised, then the needs of the various populations involved must be understood, including any sociocultural sensitivities or personal preferences [23]. One such opportunity to understand more of the need for peer support in screening for CYP is offered by the recent introduction and development of screening for Type 1 diabetes (T1D) as supported by the EarLy Surveillance for Autoimmune diabetes (ELSA) study, the first formal qualitative study to understand acceptability for a national T1D general population screening programme for children in the United Kingdom [24]. The work we present here presents a secondary, post hoc content analysis of data from a series of semi‐structured interviews with parents involved in the study that explores the preferences for and potential of peer support delivered as part of any future T1D screening programme [25]. The results are presented within an analytical framework derived from the key principles of T1D peer support developed by NHSE England, including shared experience, reciprocated support and aspects of availability and inclusivity [26]. This framework allowed us to explore the preferences of parents involved in screening and place these alongside existing evidence of established peer support programmes being used for those diagnosed and living with a range of life‐altering chronic conditions.

## 2 Methods

### 2.1 Study Design

We performed a secondary analysis of parent interview transcripts from the ELSA 1 study exploring the introduction of a national screening programme for T1D. The data were analysed using a novel framework informed by the key principles of peer support recommended for T1D [26]. The protocol for ELSA has been published in the peer‐reviewed literature [24]. The initial analysis of the overall perspectives of the programme from service stakeholders and parents has also been published [25, 27]. Health and Care Research Wales granted national research ethics approval (IRAS: 294654).

### 2.2 Recruitment and Data Collection

The data used in this analysis was gathered from semi‐structured interviews with parents [24, 28] each preceded by an introductory video outlining the screening (version 6.0, 24 January 2023, IRAS: 294654). Parents with or without prior experience of diabetes and with children aged 3–13 years were eligible to take part from across England. They were invited to participate via several routes, namely, a text message issued from their GP practice, direct invitation from a member of the study team at community outreach events and advertisements placed on popular social media platforms [27]. Of those who consented, a sample was purposively selected (as far as was able) to represent various geographical regions, ethnicity, occupation and age [27].

Specifically developed topic guides were used to explore participants' experiences of T1D, the potential pros and cons of screening children for T1D, perceived strengths and weaknesses of the ELSA screening trial and the factors influencing any decision to participate. The topic guide is summarised in File S1. Although there were no specific questions on peer support, it was raised spontaneously on multiple occasions by participants. Only those parent interviews where peer support was described were used in the secondary analysis. The interviews were undertaken by I.L. and L.M.Q., a male senior research fellow with extensive experience in health services research and a female clinical research fellow and T1D clinician, respectively. They were conducted via telephone, video call or face to face according to participant preference (though only audio recordings were made). There were no prior relationships with the study participants. The audio recordings were transcribed verbatim by an approved third‐party transcription service.

### 2.3 Data Analysis

To help understand the data in the context of T1D, we developed a prototype framework informed by the six principles of peer support in the context of T1D established by NHS England (NHSE) [26]. Their development was inspired by similar initiatives in other long‐term conditions such as HIV [29]. It was produced in conjunction with those with lived experience of T1D and the registered charities Diabetes United Kingdom (DUK) (https://www.diabetes.org.uk/) and the Juvenile Diabetes Research Foundation (JDRF) (https://jdrf.org.uk/). A summary of the six principles and their definitions that informed our analysis is provided in Box 1.

Box 1 **The six principles of peer support for people with established T1D [26].**

**Table:** 

Principle	Definition
1. Shared experiences	The support and knowledge gained from sharing experiences of others living with T1D.
2. Reciprocated support	The opportunity for people to ‘give and get’ as well as to learn from one another.
3. Open, non‐judgemental	Create a safe and encouraging environment that allows people to share their experiences free from any judgement.
4. Accessible and inclusive	Ensure that support is accessible, inclusive and available for all sections of the population with T1D.
5. Person‐centred or individualised	The support should be person‐centred, taking account of each person's strengths, values, needs and feelings.
6. Integrated	Peer support needs to be complementary and working hand in hand with health and social care providers.

Two authors independently coded each transcript, I.L. and L.M.Q., originally fitting the data within each of the six themes using the best principles of directed content analysis [30]. This followed the principles of Elo and Kyngas ‘unconstrained matrix’ approach, which explicitly allows for the development and inclusion of new or emergent themes within the framework [30, 31]. The analysis proceeded with the input of the wider team with expertise in qualitative research, S.M.G. a medical sociologist, F.B. a medical ethicist and P.N. a diabetes clinician. It was consensually decided to conflate the data into three domains due to the overlap that emerged when allocating data within each of the initial six domains. The final themes were termed *Shared experiences and reciprocated support (Principles 1* + *2),* describing the support and knowledge gained from sharing experiences of others living with T1D; *Accessibility and inclusivity (Principles 3* + *4),* the importance of accessible, safe and non‐judgemental support; and *Person‐centred and integrated support (Principles 5* + *6),* how peer support should be personalised yet integrated with existing offers of health and social care. Within each of these three domains, a number of sub‐constructs emerged from the data specific to the use of peer support in screening for T1D in CYP. The final framework including domains and constructs is presented and defined alongside the context relating to screening T1D in Table 2.

## 3 Results

### 3.1 Characteristics of Participants and Interviews

The total number of parents interviewed was 38 across 33 interviews. Of these, peer support was explicitly discussed by participants and subsequently explored by interviewers in 29 of 33 interviews (*n* = 33 parent participants with 4 interviews consisting of two parents each). A total of 13 out of 33 (40%) parents belonged to an ethnic minority group, and 16 out of 33 (52%) had personal/family experience with diabetes. All interviews were conducted before screening participation. The characteristics of the participants are summarised in Table 1. A total of 25 out of 33 (76%) of the parent interviews were video calls and the remainder 8 out of 33 (24%) telephone interviews. The median duration of the parent interviews was 54 min (range 33–87 min).

**Table 1:** Characteristics of parent participants.

*Demographics of included cohort n* = *33 (demographics of whole cohort n* = *38)*				
Mother		Father		
27 (29)		8 (9)		
*Age of parent in years* a				
25–29	30–34	35–39	40–44	45+
4 (6)	4	13	7 (8)	3
*Number of children per family (n* = *29 families)*				
1	2	3		4+
7 (8)	15 (17)	4 (5)		3
*Parental ethnicity*				
Afro‐Caribbean	Arabic	Asian	Mixed/multiple	White British
3 (6)	6	2	4	20
*Experience of diabetes*				
T1D—first‐degree relative	T2D in any family member	Friend with T1D	Unknown	None
10 (12)	4	1	1	19 (20)

^a^ Unknown for three.

### 3.2 Qualitative Results

The data are described within the analytical framework as summarised in Table 2. This contains a definition of each domain, the supporting constructs and their presentation in the context of screening for T1D. Below, we include example quotes within each construct, with participants identified by their code number (beginning with P), relationship to the CYP who would be screened and their prior experience of T1D, that is, first‐degree relative of someone with T1D (FDR or non‐FDR).

**Table 2:** Summary of preferences for peer support for T1D screening.

Peer support domain	Definition	Constructs	Preferences for T1D CYP screening
Shared experience and reciprocated support	The reciprocal support and knowledge gained from sharing experiences of others living with T1D.	Coming to terms with the result (e.g., managing emotions and initial concerns)	The shock of receiving a positive result was described and parents expressed the potential need for questions to be answered regards the consequences for the CYP's life.
		Provision of emotional support and understanding	Parents describe their potential need for reassurance from others going through or having previously faced the same experience. This would help the parent better support the assimilation of the diagnosis supported ongoing networked support to reduce feelings of isolation.
		Providing information according to individual needs and preferences	Parents described their preference for practical information that would enable preparedness; preferences for information that is accessible and tailored to varying health literacy levels.
Accessibility and inclusivity	Creating an accessible, safe and non‐judgemental environment to share experiences sensitive to the sociocultural characteristics of participants	Developing communities online and in‐person	There was a shared need amongst parents for a sense of kinship and belonging, and a safe space to share their experiences; here were diverse preferences in terms of the specific nature and mode of peer support.
		Availability	Availability was key and parents sought peer support on demand, out of hours and at the time required. They also expressed a preference for ongoing peer support throughout the life course of the child (e.g., from at‐risk to symptomatic T1D)
Person‐centred and Integrated	Support should be personalised to individuals but also complementary and integrated with the existing support of health and social care providers.	Parent and child‐centred	Peer support should adopt a person‐centred approach and be tailored to the unique needs and preferences of each individual.
		Integration with formal healthcare and social support	Peer support was viewed as complementary to clinical monitoring, and a way of signposting to or facilitating access to health and care services.

### 3.3 Shared Experiences and Reciprocated Support

#### 3.3.1 Coming to Terms With the Result

In the instance of a positive result, parents with and without lived experience of T1D described their fear of the implications of living with diabetes and the risk of complications. Out of these concerns emerged a need to seek reassurance that a future life with T1D is ‘manageable’. As one mother questioned:

How likely is this to impact on my child's quality of life or in what ways will it impact on the quality of life? Is it potentially life limiting, is it potentially lifestyle limiting, what will they be able to do, what won't they be able to do? How will it impact on their daily routine, will it change over time, will it deteriorate, will it improve over time or is it constant?

(P24, non‐FDR mother)

#### 3.3.2 Provision of Emotional Support and Understanding

After receiving a positive screening result, parents described how they would find reassurance in knowing there was support from those who were going through the same screening journey.

I suppose if it is a ‘high risk’ [result] it's knowing is there a group of the parents that can meet? Is there some sort of support network, informal support network that can be supported by yourselves from the education session? Just so that then, you've got someone [else] who is going through it.

(P4, non‐FDR mother)

The importance of gaining an understanding of the condition and the emotional support needed to process the result was apparent. One parent described how it was essential that they had come to terms with the result if they were going to support their CYP with pre‐T1D.

If you're prepared for it you have that early acceptance yourself, and I think as a parent you need that early acceptance, you need that time to process it all before you then drop everything on the plate of your child and go right this is your new life.

(P26, mother of a child with T1D)

Parents suggested that CYPs who have tested positive might be better prepared by facilitated networking with those with established T1D. In this way, they might ‘normalise’ the associated risk of living with T1D, reduce feelings of isolation and gently introduce them to the management requirements of diabetes that would become part of their future routine.

Perhaps if they have groups of people around his own age, like a support group or something, or people that can come out say, ‘Oh yes I have this as well, but I am living a normal life, I am doing this, I am in school, I am studying that’.

(P1, non‐FDR mother)

#### 3.3.3 Providing Information According to Individual Needs and Preferences

The education parents receive from healthcare providers following their CYPs' T1D diagnosis focusses on glucose monitoring, carbohydrate counting and insulin dosing [32]. Following a pre‐T1D diagnosis, parents wanted to speak to those with lived experience to gather hints and tips, learn what is not in the ‘rulebooks’ and support positive coping behaviours.

I would want reassurance [from parents of CYP with T1D] that I don't need to wrap her up in cotton wool, nothing needs to change for now, here's the information that you need to be looking out for in regard to symptoms, but don't let this consume your life.

(P12, mother)

Parents felt that support might be offered by peers at different stages of diagnosis and onset that can be accessed according to individual preference. Similarly, it was suggested that the provision of information might be staggered so as not to overwhelm parents in the early stages. As one parent explained:

I think if you can have various different sessions for the different groups of people based on their risk then that will make it more specific to them—and maybe not give them all the information [at once], you give [it] them in a different format so they can have it at their own leisure.

(P3, mother with T1D)

In particular, two parents expressed their frustration at ‘oversimplified’ resources that neither serve those with lower health literacy levels nor facilitate access to the scientific details for those seeking it. They felt written peer support resources (e.g., blogs, forums, websites) should aim to cover broad health literacy levels.

But actually there are many parents who work in laboratory specialties, research, stats, maths, and I think … and yes there are also many parents who don't speak English, can't read, but I think when you try and fix everybody with one information thing that just dumbs it all down.

(P08, non‐FDR mother)

### 3.4 Accessibility and Inclusivity

#### 3.4.1 Developing Communities Online and In‐Person

Some parents valued the convenience and accessible nature of online peer support, while others saw value in face‐to‐face contact. In both cases, the important aspect was establishing that there was a community of people going through the same or similar challenges:

I didn't realise there was a wide community of type 1 diabetics out there. I have been hooked ever since, and it's like somebody at the door and behind it there was a tribe that was waiting for me to walk in. That feeling of finding acceptance, no judgement—sorry I am getting a bit emotional.

(P10, mother with T1D)

#### 3.4.2 Availability

Parents thought peer support should be made available early on and throughout the progression from ‘at‐risk’ to symptomatic onset and beyond. They felt peer support should be used to signpost to other high‐quality resources and support systems, for example, psychological support. The use of international online peer support was cited by one parent as being particularly useful due to its potential availability over the course of 24 h:

So the self‐help groups such as Overeaters Anonymous, there's something there that's on‐demand, so if something gets the better of them at midnight they can go online to a meeting, Australia is just waking up, and they have got an English speaking meeting. It is there and it's at their disposal, and it proved quite well.

(P12, mother)

### 3.5 Person‐Centred and Integrated

#### 3.5.1 Parent and Child‐Centred Support

In considering the development of self‐management in CYP, one mother described her preference for finding parents experiencing a similar situation.

I think that would be really important to find other parents going through something similar with a child of similar age, that always really helps. And what support there would be for them [children]. Because as everyone says each of our three children are really different from each other, and they would all deal with it in quite a different way.

(P24, non‐FDR mother)

#### 3.5.2 Integration With Formal Healthcare and Social Support

Parents viewed peer support as an accompaniment to clinical monitoring for children with pre‐T1D, not a replacement. Therefore, they were keen that there was a transparent route to access clinical advice if required including regular clinical follow‐up to monitor progression.

I think as long as you knew there was some kind of pathway where if you could see there was some problems happening you had a number that you could phone to see a specialist quickly that would be helpful.

(P22, non‐FDR mother)

A parent who worked in the NHS describes adopting the principles of peer support in her own clinical practice, by reassuring, empathising and supporting the individual to feel safe and free from judgement, demonstrating key similarities between effective diabetes care and peer support.

So, I would say to them, ‘I struggled sometimes myself and had to go to the British Red Cross, do you think you might need a little bit of support with some food bank?’ But if you were to ask that outright they get so offended, not everyone. But if you say, ‘Look I have been there as well,’ all of a sudden it all comes out, because I can relate to this person, they have been there.

(P12, non‐FDR mother)

## 4 Discussion

### 4.1 Summary of Findings

Parents spontaneously described the value of peer support for children with presymptomatic T1D and their families. By modifying the six principles of (T1D) peer support into three key domains, we developed an effective framework to explore various aspects of peer support in the context of CYP screening. Reflecting on the potential benefits of *Shared experience and reciprocated support of Peer Support*, parents described its value as they came to terms with a (positive) screening result, reflecting on its ability to provide emotional support, reassurance and enable access to more direct interpretable and relevant information. In considering peer support's *Accessibility and availability*, parents shared the need to establish their place in a community of similar individuals and the importance of having that support readily available, whether in person or online. Finally, in describing the need for *Person‐centred and integrated peer support*, parents described their preference for peer support that was capable of meeting a range of needs directly relevant to their and their child's needs over time, and confirmed peer support was viewed as augmenting clinical provision and not as a replacement.

In the absence of a wider evidence base describing peer support as an adjunct to screening programmes [33], below we draw on the literature describing the existing use of parental peer support in a range of long‐term conditions and how we might learn from these in developing peer support associated with screening programmes for CYP.

### 4.2 Specific Findings

#### 4.2.1 Shared Experience and Reciprocated Support

Although a positive result in a screening test rarely results in an immediate diagnosis (instead leading to further tests and diagnostic exploration), it can be equally as transformative both for participants and their families as the confirmed diagnosis of a life‐altering condition [34, 35, 36]. In both instances, similar levels of biographical disruption are experienced as life plans change alongside fundamental shifts in self‐identity [37, 38, 39]. For example, parents of a child with a confirmed serious diagnosis have expressed feelings of shock, anger, guilt and anxiety [40, 41, 42, 43], including in the instance of T1D [44, 45]. Parents we spoke to similarly described how they would be shocked by a positive screening test, and how they would likely seek immediate reassurance and support to help manage their negative emotions. This need for immediate reassurance for parents of CYP with positive screening results has been observed previously in a paediatric screening programme for cystic fibrosis [33].

Our parent participants described the emotional reassurance they might receive if belonging to a community of parents and CYPs in a similar position and where they could share their own experiences and seek emotional support. This establishment of non‐hierarchical and reciprocal relationships through peer support, including the ability to honestly express feelings and source social approval has previously been described in a number of parent‐based peer support groups of children possessing a range of chronic conditions [46, 47, 48, 49]. Specific to T1D screening, it is notable that evidence indicates that parental depression and anxiety after the first notification of a positive screening test lessens over time, highlighting the importance of the timely delivery of such support [50, 51].

Alongside this emotional support, our parent participants described how upon receiving the result, they would likely seek practical information from peers that enabled them to be better prepared for the onset of T1D. Parents of children diagnosed with T1D have previously described being overwhelmed by clinical information at the point of diagnosis [52, 53], and this has also been reported by parents of children newly diagnosed with similar life‐altering conditions [54, 55]. Regardless of the condition or disease, the comprehension of surrounding information in the early stages after diagnosis can have a significant impact on patient decision‐making and prognosis [56]. With successful assimilation, parents can more readily begin their preparation for chronic disease management, which has been shown to improve long‐term outcomes both in T1D [50] and in a range of other chronic conditions [57].

A key benefit of peer support is its ability to provide relevant understandable information and practical strategies for illness management [58, 59, 60]. There are multiple examples of this in peer support programmes developed for adult populations [61, 62, 63]. Peer support has similarly led to the acquisition of new knowledge and improved planning amongst parents of children with a variety of long‐term conditions [48] including T1D [64].

#### 4.2.2 Accessibility and Inclusivity + Availability

Our parent participants described the potential benefits of both in‐person and online peer support formats in establishing kinship, with online groups noted for their potential to provide 24 h synchronous interactions. There is existing evidence of how online peer support can increase availability and accessibility, particularly amongst CYPs in various long‐term conditions [65, 66]. There is also evidence that online access allows individuals to choose when to engage and to react in private [67]. The potential benefits of in‐person groups were also described by our participants, including those with pre‐symptomatic CYP and parents of CYP successfully living with T1D.

Despite these preferences, it is important that the development of peer‐support groups, whether digital or in‐person, is carefully managed in order to sustain the group dynamic and retain a shared spirit of altruism and advocacy [68, 69]. There are potential harms of such contact when it lacks appropriate moderation, including sharing misinformation [70], confusion between formal advice and personal experience [71, 72] and informal judgements of asymptomatic individuals as not being relevant members [73]. Even where the groups are moderated, membership can lower self‐esteem as a result of belonging to a ‘stigmatised’ group or through comparisons between group members on the adequacy of their ability to ‘cope’ [74, 75].

#### 4.2.3 Person‐Centred and Integrated Support

Participants were keen to point out that despite the value of peer support, they felt it equally important to maintain regular clinical contact. However, attempts at integrating peer support into clinical teams have proven problematic with peer supporters and champions citing disinterest from clinical staff and conflicts of loyalty as frequent barriers [76, 77, 78]. To help address these issues, it has been suggested that health leaders should adopt a more gradual approach to integrating peer support, linking it to more commonly understood objectives, such as patient‐centred care [78]. It is also important to consider how more formal integration with mainstream healthcare is perceived by those the support is trying to reach. This is particularly important considering the previously recognised advantage of peer support engaging those otherwise suspicious of mainstream care [79, 80].

Ultimately future peer support programmes to support CYP screening for T1D or other conditions and age groups would ideally be co‐designed with peer supporters [20] and the community they are expected to serve [68]. This should lead to peer support programmes able to meet the evolving needs of parents and CYP [81] and maximising dissemination and engagement [26, 82].

### 4.3 Strengths and Limitations

This is the first time that we are aware that qualitative interview data has been used to report the perceived value of peer support for parents and participants of a CYP screening programme for any condition. Our content analysis used a framework established by the key principles of peer support developed for those with diagnosed T1D. However, it also demonstrated the universality of peer support principles, as it allowed us to capture and position the preferences for its design that we uncovered in the context of existing evidence of established peer support programmes [26, 30].

Our sample consisted of an ethnically diverse cohort of parents who raised peer support spontaneously and the secondary analysis was completed within the recommended 12 months of the primary analysis, which minimised the risk of changes to social, cultural or political norms and their potential influence over the data [83]. We acknowledge that the work presents hypothetical preferences of parents (many of whom were middle class and may tend to favour peer support) and these may change in the instance of an actual positive result. However, their perspectives on the need for structured practical and emotional support mirror the reported evidence of established peer support programmes for patients and their families in a range of life‐altering health conditions.

Existing research on peer support in screening has focussed on its use in increasing uptake [84]. However, the uncertainty of a screening test (effectively living between health and illness until symptomatic onset) described by our participants and witnessed in other childhood screening programmes [73] warrants careful consideration in any future peer support programme for CYP screening. This work is now underway for CYP screening for T1D through the ELSA and EDENT1FI studies [24, 85].

## 5 Conclusions/Implications for Practice

Screening programmes for CYP appear to offer unique challenges for those designing peer support, such as potentially lengthy periods of monitoring for symptoms, the impact of risk on the child and when and how that might be communicated. This work indicates how many of these preferences for the content and modality of peer support in T1D screening are shared by patients of all ages and their families receiving a range of life‐altering diagnoses. These include opportunities to share experiences, reduce anxiety and isolation and facilitate peer‐based learning throughout the life course. This universality suggests that future work developing peer support in a range of CYP screening programmes can be informed by existing evidence and experience. Ultimately, however, their precise content should be tailored to the specfic programme, and the needs and prognosis of the condition being screened.

## Author Contributions

**Ian Litchfield:** conceptualisation, methodology, writing–original draft, writing–review and editing, formal analysis, investigation. **Lauren M. Quinn:** conceptualisation, methodology, formal analysis, data curation, writing–review and editing, investigation. **Felicity Boardman:** methodology, writing–review and editing, formal analysis. **Olga Boiko:** writing–review and editing. **Parth Narendran:** writing–review and editing, supervision, funding acquisition, formal analysis. **Shivam Choundhary:** writing–review and editing. **Naga Setti:** writing–review and editing. **Veer Sheth:** writing–review and editing. **Sheila M. Greenfield:** methodology, formal analysis.

## Ethics Statement

Health and Care Research Wales granted national research ethics approval (IRAS: 294654).

## Consent

Written informed consent was obtained from all participants including permission for their data to be used in accordance with that consent for the use of this publication.

## Conflicts of Interest

The authors declare no conflicts of interest.

## Supporting information

Supporting information.

## Data Availability

The data are not publicly available due to their containing information that could compromise the privacy of research participants.
