# Erratum to “Preferences for Peer Support Amongst Families Engaged in Paediatric Screening Programmes: The Perspectives of Parents Involved in Screening for Type 1 Diabetes in Children Aged 3–13”

**DOI:** 10.1111/hex.70115

**Published:** 2024-11-27

I. Litchfield, L. M. Quinn, F. Boardman, et al., “Preferences for Peer Support Amongst Families Engaged in Paediatric Screening Programmes: The Perspectives of Parents Involved in Screening for Type 1 Diabetes in Children Aged 3–13,” *Health Expectations* 27, no. 4 (August 2024): e70007, https://doi.org/10.1111/hex.70007.

Surname of the sixth author needs changing from Choundhary to Choudhary.

We apologize for this error.

