# Supplementary material for: Preferences for Peer Support Amongst Families Engaged in Paediatric Screening Programmes: The Perspectives of Parents Involved in Screening for Type 1 Diabetes in Children Aged 3–13
Source: Health Expect. 2024 Aug 27;27(4):e70007. doi: 10.1111/hex.70007 (PMC11348000; doi:10.1111/hex.70007)
Supplement: Supplementary file 1 — Supporting information. [file HEX-27-e70007-s001.docx]

**Supplementary File 1 – Topic guide**

Sections and sub-sections included in the interview topic guide for parents.

| **Section** | **Sub-sections** |
| --- | --- |
| 1. Video presentation outlining the proposed ELSA screening trial | *-Initial thoughts*  *-Any questions* |
| 1. Understanding of T1D | *-Lived experience of diabetes*  *-Diabetes in their wider social network*  *-Effect on day-to-day life* |
| 1. Views about screening children for T1D | *-Thoughts and feelings on screening*  *-Reasons for or against screening children for T1D*  *-Perceived benefits and harms of screening*  *-Reservations or concerns about screening*  *-Thoughts on monitoring children at-risk and effect on decision to screen*  *-Thoughts on prevention trials for children at risk and effect on decision to screen*  *-Thoughts on a licensed treatment to delay onset and effect on decision to screen*  *-Parent making the decision on behalf of their child* |
| 1. Practicalities and mechanics of a screening trial | *-Thoughts on the screening process*  *-Necessary information to decide whether to participate*  *-Reaction to screening test and further testing*  *-Preferred screening location*  *-Preferences for communicating results*  *-Reaction to a low-risk, intermediate or high-risk screening result*  *-Preferences for education*  *-Thoughts on opt-out screening*  *-Perceived implications of screening*  *-Decision to screen or not* |
